# Associations between one-carbon metabolism and valproic acid-induced liver dysfunction in epileptic patients

**DOI:** 10.3389/fphar.2024.1358262

**Published:** 2024-02-23

**Authors:** Jingwei Zhu, Zhe Wang, Xiaotong Sun, Dan Wang, Xinbo Xu, Liping Yang, Jiangdong Du, Zhimei Zhou, Yanhua Qi, Linfeng Ma

**Affiliations:** ^1^ Department of Clinical Laboratory, Qingdao University Medical College Affiliated Yantai Yuhuangding Hospital, Yantai, China; ^2^ School of Life Science, Jilin University, Changchun, China

**Keywords:** Valproic acid, one-carbon metabolism, MTHFR A1298C, MTHFR C677T, homocysteine, oxidative stress, liver dysfunction

## Abstract

Valproic acid (VPA) has been widely used as an antiepileptic drug for decades. Although VPA is effective and well-tolerated, long-term VPA treatment is usually associated with hepatotoxicity. However, the underlying mechanisms of VPA-caused hepatotoxicity remain unclear. In this study, a total of 157 pediatric patients with epilepsy were recruited and divided into normal liver function (NLF, 112 subjects) group and abnormal liver function (ABLF, 45 subjects) group. We observed that *MTHFR* A1298C and *MTHFR* C677T variants may be linked to VPA-induced liver dysfunction (*p* = 0.001; *p* = 0.023, respectively). We also found that the *MTHFR* A1298C polymorphism was associated with a higher serum Hcy level (*p* = 0.001) and a lower FA level (*p* = 0.001). Moreover, the serum Hcy levels was strongly correlated with the GSH and TBARS concentrations (r = −0.6065, *P* < 0.001; r = 0.6564, *P* < 0.001, respectively). Furthermore, logistic analysis indicated that *MTHFR* A1298C/C677T polymorphisms and increased Hcy concentrations may be risk factors for VPA-induced liver dysfunction. These results suggested that individual susceptibility to VPA-induced liver dysfunction may result from *MTHFR* A1298C/C677T polymorphisms and increased Hcy levels. This study may be helpful for the prevention and guidance of VPA-induced liver dysfunction.

## Graphical Abstract

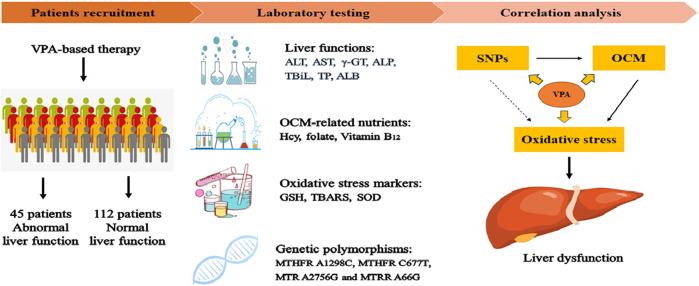

## Introduction

Epilepsy, one of the most common central nervous system disorders, affects more than 70 million people worldwide (Thijs et al., 2019). Epilepsy is usually characterized by the recurrence of unprovoked seizures, leading to neurological injury as well as psychosocial and cognitive consequences. For most epileptic patients, anti-epileptic drugs (AEDs) treatment is a priority modality. Valproic acid (VPA), a broad-spectrum AED, has been prescribed predominantly for the treatment of epilepsy and bipolar disorder for decades (Mishra et al., 2021). Although VPA was confirmed to be effective and well-tolerated, long-term VPA treatment is usually accompanied by hepatotoxicity (Guo et al., 2019; Ezhilarasan and Mani, 2022) However, the underlying mechanism for VPA-induced hepatotoxicity is not fully understood.

**FIGURE 1:**
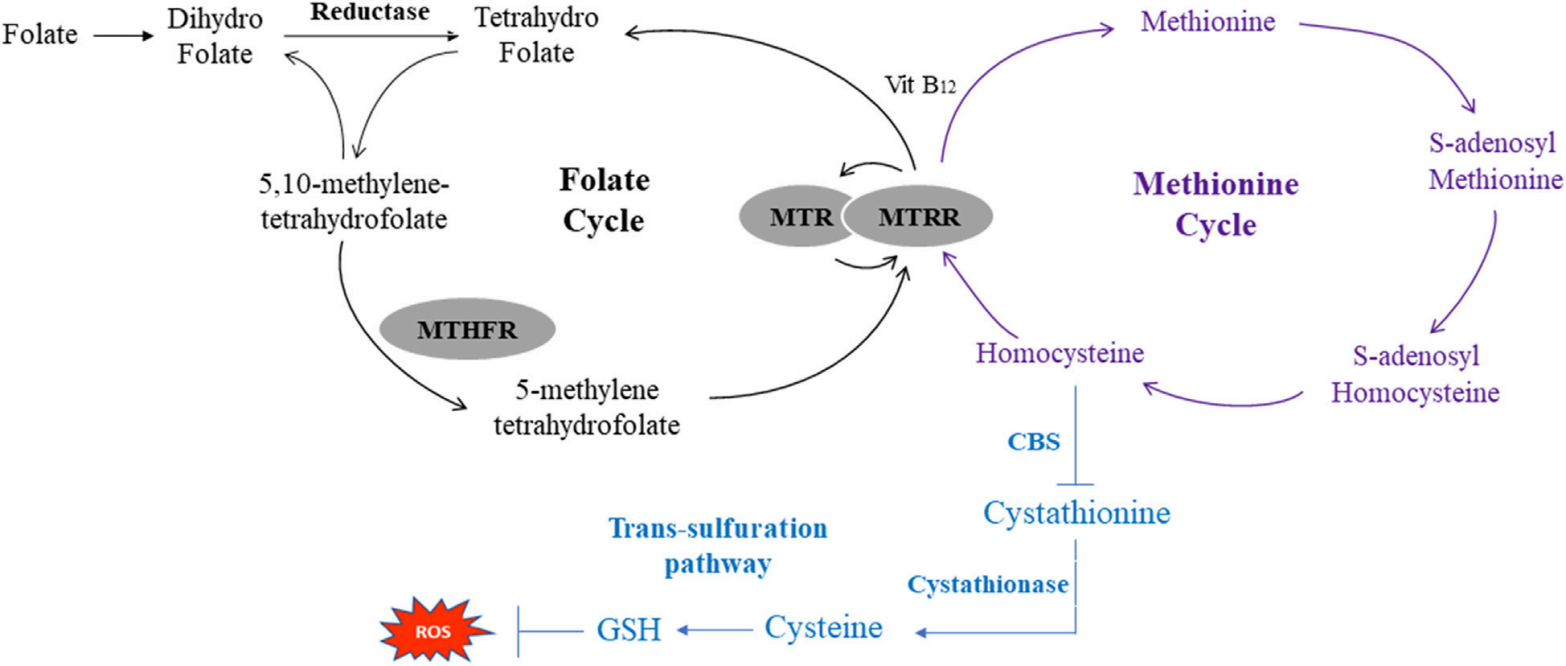
Schematic of One-Carbon metabolism related folate cycle, methionine cycle and transsulfration pathway. MTHFR, methylene tetrahydrofolate reductase; MTR, methionine synthase; MTRR, methionine synthase reductase CBS, cystathionine beta-synthase; ROS, reactive oxygen species.

Accumulating evidence indicates that one-carbon metabolism (OCM) is involved in the progression of alcoholic liver disease (ALD) and nonalcoholic fatty liver disease (NAFLD) (Tsuchiya et al., 2012; Radziejewska et al., 2020). One-carbon (1C) metabolism, mediated by folate cofactors and co-substrates [Vitamin B_6_ (VB_6_), Vitamin B_12_ (VB_12_)], serves multiple biological processes (Ducker and Rabinowitz, 2017). In detail, folate and methionine cycles are included in the progression of OCM (Raghubeer and Matsha, 2021). Specifically, dietary folate acid (FA) is converted and reduced to tetrahydrofolate (THF). THF is converted to 5,10‐methyleneTHF and then to 5‐methylTHF via methylene tetrahydrofolate reductase (MTHFR) in the folate cycle (Petrone et al., 2021). 5‐methylTHF is used as a methyl donor to generate methionine and its subsequent products [such as S-adenosyl Methionine, S-adenosyl homocysteine (Hcy) and Hcy] in the methionine cycle. This reaction is catalyzed by methyltransferase (MTR) and methionine synthase reductase (MTRR) with VB_12_ serving as a cofactor. Meanwhile, Hcy can also entre the transsulfuration pathway to synthesize glutathione (GSH) to defend against redox reactions (Figure 1) (McBean, 2012). However, there are limited data about its relationship with VPA-induced liver disease.

Previous study indicated that VPA treatment disrupted the homeostasis of OCM in epileptic patients (Ni et al., 2018a). Additionally, another study indicated that patients underwent long-term VPA treatment were more susceptible to OCM dysmetabolism than general population (Ni et al., 2017). However, due to the complicated compositions of OCM, the mechanism in susceptibility to VPA-induced liver dysfunction remains elusive. On the one hand, OCM-related genetic polymorphisms, including *MTHFR* (C677T, rs1801133) and *MTR* (A2756G, rs1805087) were associated with DNA methylation in VPA-treated patients (Vurucu et al., 2008; Ni et al., 2018b). Moreover, *MTHFR* A1298C and *MTRR* A66G were also reported to be involved in the progression of liver diseases (Kasapoglu et al., 2015; Peres et al., 2016). On the other hand, VPA therapy (once or long-term) altered the levels of OCM-associated nutrients (FA, Hcy and VB_12_) (Gidal et al., 2005; Zhu et al., 2022). Taken together, OCM-related genetic variants and nutrient levels are highly correlated with VPA treatment in epileptic patients. However, to the best of our knowledge, there is no direct evidence supporting the association between OCM and VPA-induced liver dysfunction. Alternatively, numerous studies have demonstrated that Hcy can induce oxidative stress in molecular and cellular aspects (Perna and De Santo, 2003; Scherer et al., 2011). Moreover, oxidative stress is recognized as a key inducer of the progression of VPA-induced liver dysfunction (Chang and Abbott, 2006). Based on these findings, we hypothesize that OCM-related nutrients, genetic polymorphism and oxidative stress may contribute to VPA-induced liver dysfunction, but the mechanism underlying VPA-induced liver dysfunction remain unclear.

In this study, we systematically explored the associations between OCM-related genetic polymorphisms and nutrient levels as well as oxidative stress with VPA-induced liver dysfunction in epileptic patients.

## Materials and methods

### Patients

This study included 157 patients with a diagnosis of symptomatic epilepsy based on the etiologic classification of epilepsy (Fisher et al., 2014). All patients were recruited at Yuhuangding Hospital between December 2021 to February 2023. For each patient, demographic characteristics (age, BMI, VPA dose, concomitant drug therapy and liver function) were recorded. In addition, patients were divided into a normal liver function [NLF, the levels of all liver function indicators did not exceed the upper limit of normal (ULN), 112 subjects] group and an abnormal liver function (ABLF, at least one indicators exceed two-times of the ULN, 45 subjects) group according to liver function indicators as described previously (Chen et al., 2019). Meanwhile, patients with the following conditions were excluded from this study: 1) pre-existing of ABLF; 2) hyperhomocysteinemia; 3) other potential causes of liver disease including hepatitis A/B/C, HIV- positive or metabolic disease 4) smoking, alcohol or drug abuse; 5) less than 3-month of VPA-based treatment.

This study was approved by the Ethics Committee of the Affiliated Yuhuangding Hospital of Qingdao University (No. QDU-HEC-2023108). Informed consents were signed by patients or their legal guardians. Sample handling and data analysis protocols followed the clinical principles of Yuhuangding Hospital.

### Blood collection and laboratory assays

After fasting overnight for approximately 10 h, venous blood was collected from each patient using sterile clot activator tubes. Blood samples were centrifuged at 3500 rpm for 5 min and then analyzed within 30 min or stored at −80 °C for later analysis.

Liver function indicators, such as alanine aminotransferase (ALT), aspartate aminotransferase (AST), alkaline phosphatase (ALP), gamma-glutamyltransferase (γ-GT), total bilirubin (TBiL), total protein (TP), albumin (ALB) and Hcy were determined by an automatic biochemistry analyzer (Au5800, Beckman Coulter, United States). The FA and VB12 concentrations were determined by chemiluminescent immunoassay using automated techniques (e601, COBAS, Roche, Germany) based on the protocols given by the manufacturer.

### Quantification of VPA concentrations

In this study, patients underwent more than 3 months of VPA-based therapy to ensure a steady-state VPA concentration. Ethylenediaminetetraacetic acid (EDTA)-2Na tubes were used to obtain venous blood samples from each patient. To measure the steady-state VPA concentration, blood samples were collected just before the last VPA administration. Subsequently, the VPA concentration was quantitatively measured using an automatic fluorescence immunoassay system (Abbott, Chicago, United States) as described previously (Ma et al., 2019).

### DNA extraction and genotyping analysis

A total of 5 mL of peripheral blood was obtained from each participant using an EDTA tube and stored at −20 °C. Genomic DNA was extracted by DNA Extraction Kit (OMEGA, Norcross, United States) according to the manufacturer’s protocols. The *MTHFR* A1298C, *MTHFR* C677T, *MTR* A2756G and *MTRR* A66G polymorphisms were detected directly by DNA sequencing after PCR amplification using an automatic genetic analyzer (Applied Biosystems, United States). Details of genetic polymorphisms and sequences of primers used for genotyping procedures are presented in Supplementary Table S1.

### Measurement of oxidative stress and antioxidative parameters

The concentration of GSH was quantitatively measured according to the protocol given by Chien’s study (Chien et al., 2016). Briefly, 10 μL of serum was mixed in 990 μL cold phosphate (10 μM)/EDTA (5 μM) buffer. Then, add 1 volume ice trichloroacetic acid (TCA) into 5 volumes of sample, and centrifuge samples at 12,000 g for 5 min. Next, the supernatant was neutralized by NaHCO_3_ and measured spectrophotometrically at 405 nm. Serum SOD activity and TBARS concentrations were determined by Beckman Coulter ACCESS ^®^ (Brea, CA, United States) and suitable kits according to the manufacturer’s instructions.

### Statistical analysis

The statistical analysis in this study was performed using SPSS (version 20.0; IBM, United States). For demographic characteristic analysis, Student’s t-test was used to evaluate significant differences between the NLF and ABLF groups. The statistical significance of the differences in VPA concentration and concomitant drug were determined by Student’s t-test or Fisher’s exact test. Comparisons of genotype frequencies were performed by χ2 (chi-square) tests. Logistic regression was used to evaluate the risk factors for VPA-induced liver dysfunction. Data are presented as the mean ± standard deviation. *p*-value less than 0.05 indicated a statistically significant difference.

## Results

### Demographic characteristics of the epileptic patients

This study included 157 pediatric patients (aged: 6.93 ± 6.02 years in the ABLF group and 6.16 ± 4.80 years in the NLF group) with VPA-based therapy. Patients were divided into ABLF and NLF groups based on the results of liver function tests. The demographic characteristics of ABLF and NLF patients are summarized and listed in Table 1. Patients in ABLF group exhibited significantly higher ALT, AST, γ-GT and TBiL levels than those in NLF group (*P* < 0.05). Moreover, no differences in age, BMI, TP, ALB or globulin (GLO) levels were observed between ABLF and NLF groups (Table 1).

**TABLE 1:** Demographic characteristics and biochemical indicators of patients.

Demographic characteristics	ABLF	NLF	*p*-value
Number of patients	45 (28.7%)	112 (71.3%)	-
Age (years)	6.93 ± 6.02	6.16 ± 4.80	0.449
Height (cm)	120.11 ± 39.88	110.88 ± 32.48	0.172
Body weight (kg)	29.80 ± 15.80	25.28 ± 13.77	0.098
BMI (kg/m^2^)	21.05 ± 6.91	21.14 ± 10.88	0.956
TP (g/L)	68.67 ± 8.21	67.01 ± 5.52	0.215
ALB (g/L)	42.78 ± 8.21	42.44 ± 4.55	0.796
GLO (g/L)	25.89 ± 7.15	24.56 ± 5.11	0.192
TBiL (μmol/L)	9.79 ± 6.34	6.90 ± 2.76	**0.005**
γ-GT(U/L)	57.76 ± 71.67	20.04 ± 15.29	**0.001**
ALP (U/L)	188.98 ± 125.61	181.12 ± 64.48	0.691
ALT (U/L)	80.96 ± 42.79	13.66 ± 7.09	**<0.001**
AST (U/L)	92.91 ± 64.40	26.41 ± 8.97	**<0.001**

Data are presented as Mean ± S.D., The bolded data indicated *p* < 0.05.

Reference ranges: TP, 65–85 g/L, ALB, 40–55 g/L, TBiL = 3.4–20.5 μmol/L, γ-GT, 9–64 U/L, ALP, 40–375 U/L, ALT, 0–40 U/L, AST, 5–34 U/L.

### Comparisons of VPA concentrations, concomitant drugs and OCM-related nutrition levels between the ABLF and NLF groups

In this study, all patients underwent VPA-based therapy (monotherapy or polytherapy). We recorded the VPA dose and concomitant drug used for each patient in ABLF and NLF groups. Moreover, we analyzed the VPA concentration by an automatic fluorescence immunoassay system. As shown in Table 2, there were no significant differences in VPA dose, VPA concentration or adjusted VPA concentration between two groups, suggesting that neither the VPA dose nor the concentration may be directly associated with VPA-induced liver dysfunction.

**TABLE 2:** Comparisons of VPA concentrations, concomitant drugs and OCM-related nutrients in ABLF and NLF groups.

Items	ABLF (n = 45)	NLF (n = 112)	*p*-value
VPA concentration (mg/mL)	67.37 ± 27.17	59.78 ± 22.99	0.103^a^
VPA daily doses (mg/kg)	27.23 ± 21.69	26.24 ± 27.75	0.831^a^
Adjusted VPA concentration ((mg/mL)/(mg/kg))	3.16 ± 2.60	3.93 ± 3.21	0.159^a^
Carbamazepine	2 (4.4%)	4 (3.6%)	0.554^b^
Clonazepam	0 (0%)	2 (1.8%)	0.508^b^
Lamotrigine	2 (4.4%)	5 (4.7%)	0.679^b^
Levetiracetam	1 (2.2%)	3 (2.7%)	0.676^b^
Topiramate	2 (4.4%)	4 (3.6%)	0.554^b^
Oxcarbazepine	0 (0%)	1 (0.9%)	0.713^b^
Homocysteine (μmol/L)	9.43 ± 2.50	7.04 ± 1.37	**<0.001** ^a^
Folate (ng/mL)	14.82 ± 2.72	19.95 ± 2.37	**0.001** ^a^
Vitamin B12 (ng/L)	378.11 ± 182.28	445.54 ± 158.62	**0.022** ^a^

Data are presented as Mean ± S.D.

^a^ Statistical significance was determined by Student’s t-test for independent samples.

^b^ Statistical significance was determined by the Fisher’s exact test.

In addition, carbamazepine (ABLF: 2, NLF: 4), clonazepam (NLF: 2), lamotrigine (ABLF: 2, NLF: 5), levetiracetam (ABLF: 1, NLF: 3), topiramate (ABLF: 2, NLF: 4) and oxcarbazepine (NLF: 1) were combined with VPA therapy. However, no significant association was found between concomitant drugs and liver dysfunction in the ABLF or NLF groups (Table 2).

### Comparisons of OCM-related nutrition levels between the ABLF and NLF groups

To evaluate the association between OCM and VPA-induced liver dysfunction, we first analyzed the levels of OCM-related nutrients in the ABLF and NLF groups. As shown in Table 2, there was a clear separation between the two groups. Patients in ABLF group had significantly higher Hcy level than that in NLF group (*P* < 0.05). In contrast, the FA and VB_12_ concentrations were significantly lower in ABLF group than those in NLF group (*P* < 0.05).

**FIGURE 2:**
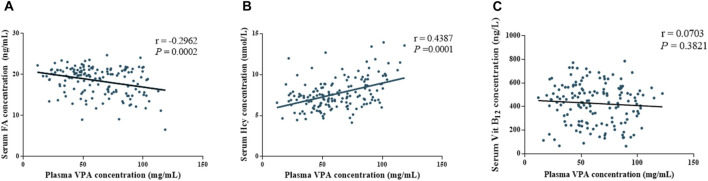
Associations between plasma VPA concentrations and OCM-related nutrition levels of all subjects (ABLF and NLF groups). Relationships between **(A)** plasma VPA concentrations and folate levels. **(B)** plasma VPA concentrations and homocysteine levels. **(C)** plasma VPA concentrations and Vitamin B_12_ levels.

Then, we explored the effect of VPA treatment on OCM, correlation analysis was performed and exhibited in Figure 2. As shown in Figure 2, there was a significant negative correlation between the plasma VPA concentration and FA concentration (r = −0.2962, *P* < 0.05), and a significant positive correlation between the plasma VPA concentration and Hcy concentration (r = 0.4387, *P* < 0.05). Interestingly, although the VB_12_ concentration displayed a separation between ABLF and NLF groups, there was no significant correlation between the plasma VPA concentration and VB_12_ concentration (*p* > 0.05). These results suggest that OCM pathway contributes to VPA-induced liver dysfunction.

### Associations between OCM-related genetic polymorphisms and VPA-induced liver dysfunction

To further elucidate the association between OCM and VPA-induced liver dysfunction, we analyzed the OCM pathway-related genetic variants (*MTHFR* A1298C, *MTHFR* C677T, *MTR* A2756G and *MTRR* A66G) in the ABLF and NLF groups. The Hardy-Weinberg equilibrium test and allele frequencies of the four genes are listed in Supplementary Table S2. The associations between the four gene polymorphisms and VPA-induced liver dysfunction are listed in Table 3. In detail, the frequencies of AC and CC genotypes in *MTHFR* A1298C were significantly higher in ABLF group than those in NLF group (48.9% *versus* 31.2%, *p* = 0.004; 15.5% *versus* 1.8%, *p* = 0.001, respectively). Meanwhile, there was a significantly increased frequency of TT genotype of *MTHFR* C677T in ABLF group when compared to NLF group (15.6% *versus* 6.3%, *p* = 0.023). However, there were no statistically significant correlations between the *MTR* A2756G or *MTRR* A66G genotype and VPA-associated liver dysfunction (*p* > 0.05, Table 3). These results suggested that *MTHFR* A1298C and *MTHFR* C677T may be risk factors for VPA-induced liver dysfunction.

**TABLE 3:** Comparisons of OCM-related genotypes frequencies between ABLF and NLF groups.

Genotypes	ABLF	NLF	Odds ratio (95% CI)	*p*-value
***MTHFR* A1298C**				
AA	16 (35.6%)	75 (67.0%)	Referent	
AC	22 (48.9%)	35 (31.2%)	1.342 (1.070–1.684)	**0.004**
CC	7 (15.5%)	2 (1.8%)	3.709 (1.088–12.637)	**0.001**
***MTHFR* C677T**				
CC	13 (28.9%)	50 (44.6%)	Referent	
CT	25 (55.6%)	55 (49.1%)	1.154 (0.951–1.402)	0.154
TT	7 (15.6%)	7 (6.3%)	1.587 (0.926–2.720)	**0.023**
***MTR* A2756G**				
AA	29 (64.4%)	76 (67.9%)	Referent	
AG	13 (28.9%)	30 (26.8%)	1.037 (0.825–1.305)	0.749
GG	3 (6.7%)	6 (5.3%)	1.281 (0.674–1.749)	0.714
***MTRR* A66G**				
AA	17 (37.8%)	42 (37.5%)	Referent	
AG	23 (51.1%)	60 (53.6%)	0.985 (0.798–1.215)	0.886
GG	5 (11.1%)	10 (8.9%)	1.068 (0.721–1.582)	0.732

The bolded data indicated *p* < 0.05.

Statistical significance was determined by the chi-square test.

### Effect of OCM-related genetic polymorphisms on nutrient levels in ABLF and NLF patients

**FIGURE 3:**
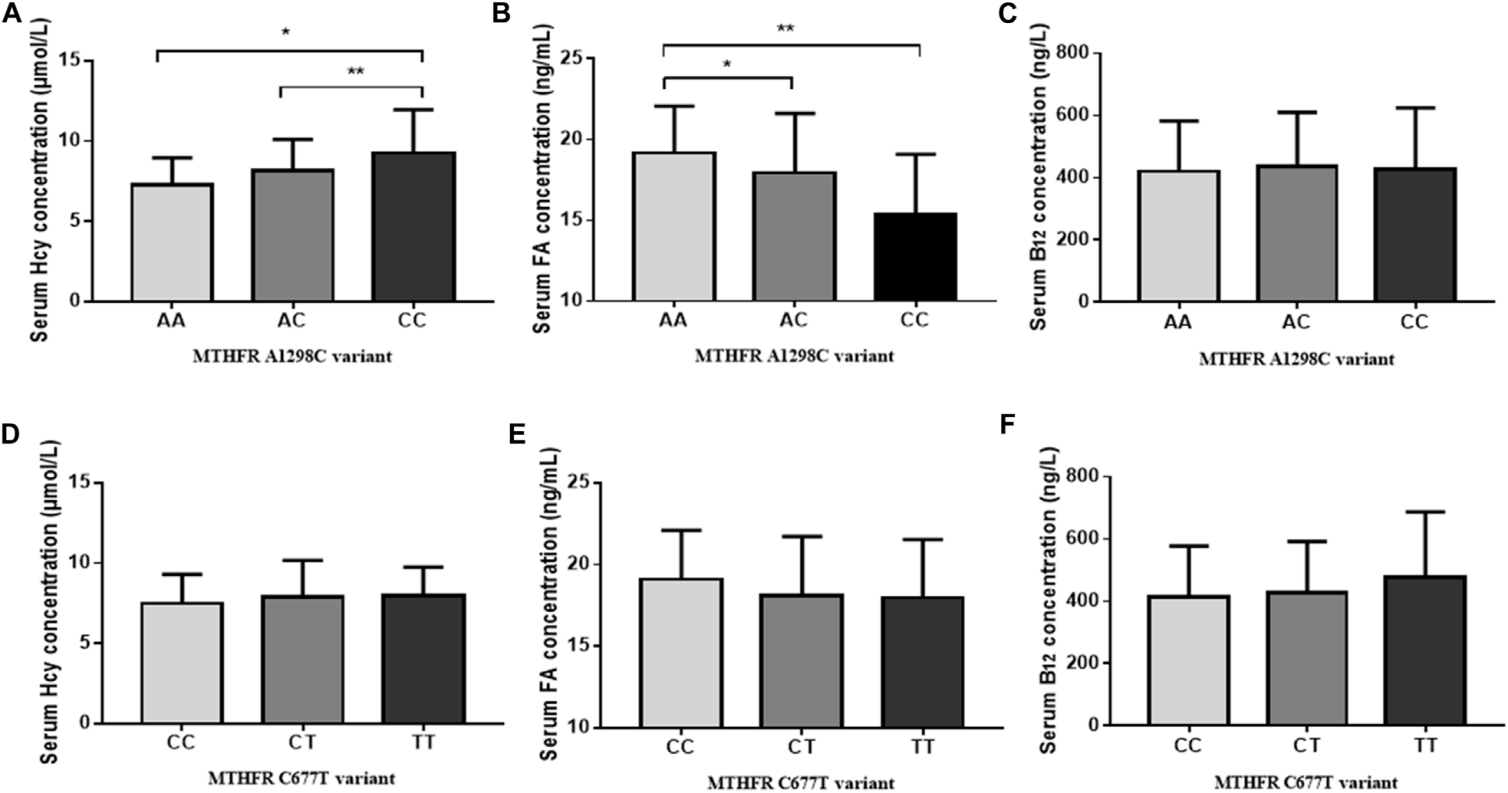
Effect of genetic polymorphisms on OCM-related nutrition levels. **(A)** *MTHFR* A1298C variant and serum Hcy levels. **(B)** *MTHFR* A1298C variant and serum FA levels. **(C)** *MTHFR* A1298C variant and serum Vitamin B_12_ levels. **(D)** *MTHFR* C677T variant and serum Hcy levels. **(E)** *MTHFR* C677T variant and serum FA levels. **(F)** *MTHFR* C677T variant and serum Vitamin B_12_ levels. *p < 0.05, **p < 0.01.

The associations between OCM-related genetic polymorphisms and nutrient levels (FA, Hcy and vitamin B_12_) in ABLF and NLF patients were analyzed. As shown in Table 4, we observed that the *MTHFR* A1298C polymorphism was associated with serum Hcy (AA: 7.25 ± 1.70; AC: 8.16 ± 1.93; CC: 9.63 ± 3.81, *p* = 0.001) and FA (AA: 19.17 ± 2.89; AC: 17.92 ± 3.70; CC: 17.97 ± 3.56, *p* = 0.001) levels. In addition, the *MTHFR* CC genotype resulted in higher Hcy levels (*P* < 0.01) and lower FA levels (*P* < 0.01) than the AA genotype (Figure 3). Interestingly, although *MTHFR* C677T showed a different genotype frequency between the ABLF and NLF groups, the differences in the *MTHFR* C677T polymorphism among OCM-related nutrient levels were not statistically significant (*p* > 0.05, Table 4; Figure 3).

**TABLE 4:** Effects of OCM-related SNPs on Hcy, Folate and Vitamin B_12_ levels in patients with epilepsy.

Items	Homocysteine	Folate	Vitamin B12
***MTHFR* A1298C**			
AA (n = 91)	7.25 ± 1.70	19.17 ± 2.89	419.89 ± 162.69
AC (n = 57)	8.16 ± 1.93	17.92 ± 3.70	436.38 ± 173.66
CC (n = 9)	9.63 ± 3.81	17.97 ± 3.56	426.85 ± 197.04
*p*-value	**0.001**	**0.001**	0.848
***MTHFR* C677T**			
CC (n = 63)	7.48 ± 1.81	19.08 ± 3.02	413.78 ± 163.00
CT (n = 80)	7.87 ± 2.30	18.10 ± 3.60	426.89 ± 164.64
TT (n = 14)	7.99 ± 1.75	7.25 ± 1.70	478.34 ± 208.33
*p*-value	0.474	0.196	0.431

The bolded data indicated *p* < 0.05.

Statistical significance was determined by ANOVA.

We further analyzed the associations between *MTR* A2756G and *MTRR* A66G polymorphisms and OCM-related nutrition levels. However, there were no statistically significant differences between *MTR* A2756G and *MTRR* A66G polymorphisms in OCM-related nutrition levels (*p* > 0.05, Supplementary Table S3).

### Associations between oxidative stress and VPA-induced liver dysfunction

Oxidative stress is well known to be involved in the progression of VPA-associated liver dysfunction (Chang and Abbott, 2006; Gai et al., 2020). Hence, we examined oxidative stress-related parameters. As shown in Table 5, a significantly higher TBARS concentration and a lower GSH concentration were observed in ABLF patients than in NLF patients (*P* < 0.05). These results demonstrated that oxidative stress is associated with VPA-induced liver dysfunction.

**TABLE 5:** Concentrations of OCM-related oxidative stress parameters in ABLF and NLF groups.

Oxidative stress parameters	ABLF	NLF	*p*-value
SOD (U/mL)	3.64 ± 2.15	3.09 ± 1.19	0.115
GSH (μmol/L)	33.90 ± 4.32	47.99 ± 4.33	**<0.001**
TBARS (nmol/mL)	2.40 ± 0.48	1.97 ± 0.24	**0.001**

Data are presented as Mean ± S.D., The bolded data indicated *p* < 0.05.

Statistical significance was determined by Student’s t-test for independent samples.

**FIGURE 4:**
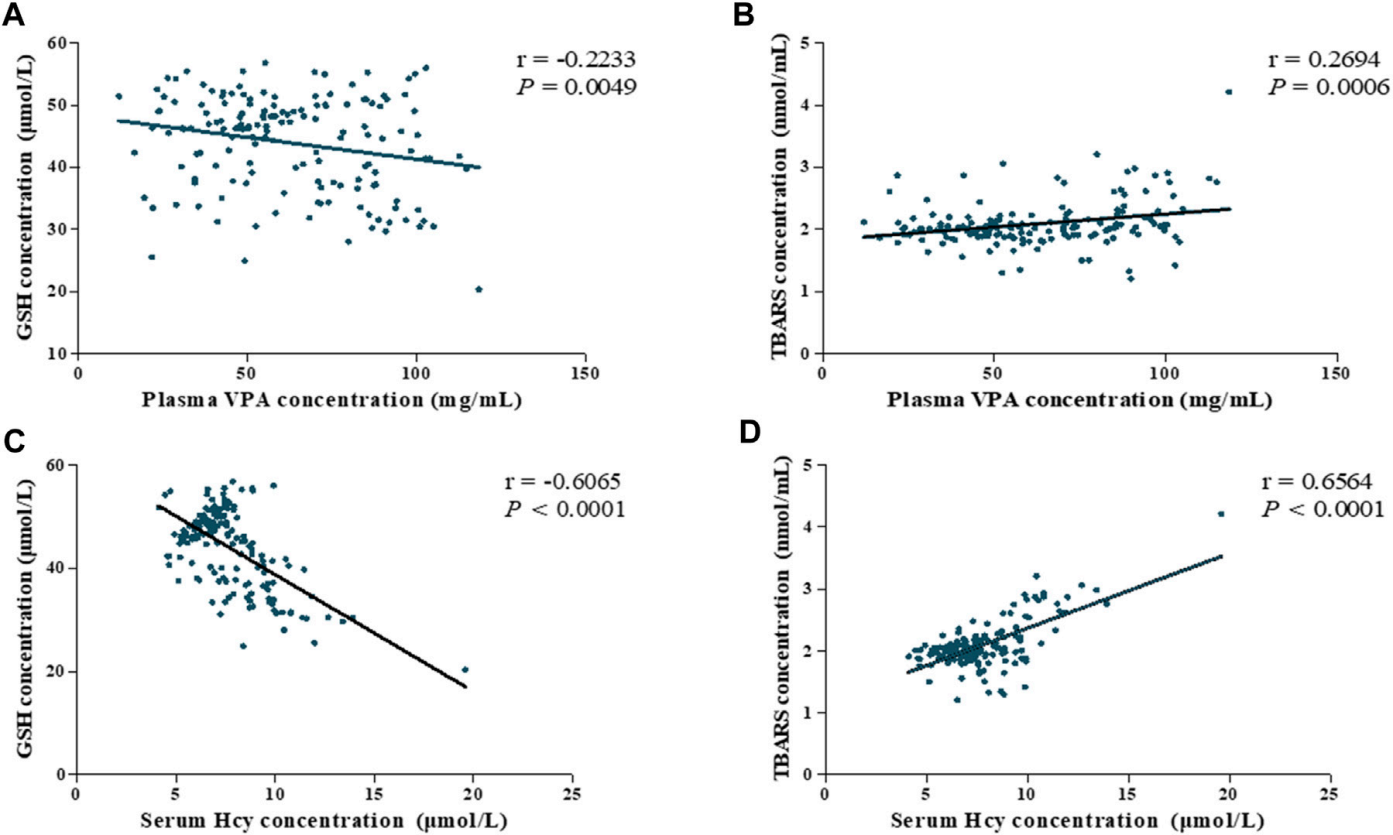
Association among plasma VPA concentration, serum Hcy levels and oxidative stress parameters of all subjects (ABLF and NLF groups). Relationships between **(A)** plasma VPA concentrations and GSH levels. **(B)** plasma VPA concentrations and TBARS levels. **(C)** serum Hcy concentration and GSH levels. **(D)** serum Hcy concentration and TBARS levels.

Considering the potential link between VPA administration and oxidative stress, we next performed correlation analysis to explore the influences of VPA concentration and oxidative stress. In this study, the VPA concentrations was significantly positively correlated with TBARS concentration (r = 0.2694, *p* = 0.006) and a significantly negative correlation with GSH concentration (r = −0.2233, *p* = 0.0049, Figures 4A,B), suggesting that the VPA concentration is associated with oxidative stress.

Importantly, Hcy is involved in redox signaling via regulating ROS generation and GSH levels (Gomez et al., 2011). For this reason, we analyzed the correlation between Hcy levels and oxidative stress. As shown in Figures 4C,D, we found that Hcy concentration exhibited highly correlated with GSH and TBARS concentrations (r = −0.6065, *P* < 0.0001; r = 0.6564, *P* < 0.0001, respectively). Based on these findings, Hcy-related oxidative stress might be involved in the pathogenesis of VPA-induced liver dysfunction.

### Analysis of risk factors for VPA-induced liver dysfunction

To further clarify the associations of OCM-related nutrients and genetic factors with VPA-induced liver dysfunction, logistic regression analysis was performed. As shown in Table 6, the *MTHFR* A1298C polymorphism (r = 1.646, *p* = 0.001), *MTHFR* C677T polymorphism (r = 1.162, *p* = 0.007) and Hcy level (r = 1.334, *p* = 0.030) exhibited a significant positive correlation with VPA-associated liver dysfunction. Moreover, there were significant negative correlations between FA, GSH concentrations and VPA-induced liver dysfunction (r = −0.602, *p* = 0.025; r = −1.258, *p* = 0.006, respectively). Interestingly, although TBARS concentrations displayed only a marginally significant association with VPA-caused liver dysfunction (*p* = 0.067), there was a 2.6-fold (95% CI: 0.693–6.436) increased risk for liver dysfunction progression. These results indicated that *MTHFR* A1298C/C677T polymorphisms and GSH/Hcy/FA concentrations may be risk factors for VPA-induced liver dysfunction.

**TABLE 6:** Logistic regression analysis of risk factors for VPA-induced liver dysfunction.

Variables	Regression coefficient	*p*-value	Exp (B)	95% Confident interval
Age	0.019	0.595	1.019	0.951–1.091
BMI	0.002	0.933	1.002	0.966–1.038
VPA doses	0.011	0.138	1.011	0.966–1.038
VPA concentrations	1.219	0.059	1.519	0.780–2.040
Adjusted VPA concentrations	−0.139	0.251	0.871	0.687–1.103
Homocysteine	1.334	**0.030**	3.802	1.135–12.658
Folate	−0.602	**0.025**	0.548	0.324–0.927
Vitamin B12	0.001	0.894	1.001	0.993–1.008
SOD	0.946	0.242	1.575	0.529–2.540
GSH	−1.258	**0.006**	0.284	0.116–0.695
TBARS	2.519	0.067	5.627	0.693–46.436
*MTHFR A1298C*	1.646	**0.001**	5.188	2.324–11.581
*MTHFR C677T*	1.162	**0.007**	3.196	1.375–7.431
*MTR A2756G*	0.135	0.738	1.144	0.519–2.525
*MTRR A66G*	−0.111	0.778	0.895	0.414–1.934

The bolded data indicated *p* < 0.05.

## Discussion

Accumulating evidence indicate that dysregulation of OCM is associated with NAFLD, but rarely reported in VPA-induced liver disease (de Carvalho et al., 2013; Fernández-Ramos et al., 2022). In this study, patients with long-term VPA treatment exhibited dysmetabolism of OCM, leading to alternations in OCM-associated nutrients and increased risk of liver dysfunction. Moreover, we demonstrated that *MTHFR* A1298C and *MTHFR* C677T polymorphisms contributed to VPA-induced liver dysfunction in patients with epilepsy. To the best of our knowledge, this study first revealed the association between OCM and VPA-induced liver dysfunction.

Patients with long-term VPA treatment are prone to OCM dysmetabolism, leading to hyperhomocysteinemia and DNA hypomethylation (Sener et al., 2006; Vurucu et al., 2008; Ni et al., 2018b). OCM is well known to be associated with chronic liver disease, but rarely reported in acute liver disease, and no report in VPA-induced liver disease (Woo et al., 2006; Tsuchiya et al., 2012; Pogribny et al., 2013). The relationship between OCM and VPA-induced liver dysfunction is unclear. In this study, ABLF patients showed lower levels of FA when compared to NLF patients (*p* = 0.001). Similarly, previous studies demonstrated that VPA treatment lowered FA levels by blocking the reuptake of folic acid, leading to a 25%–35% decrease in FA levels (Rubinchik-Stern et al., 2018; Reynolds and Green, 2020). Moreover, we also demonstrated that the levels of VB_12_ were also decreased in ABLF patients than NLF patients (*p* = 0.022). Interestingly, the concentration of VB_12_ level has a poor correlation with VPA concentration. In fact, not only VB_12_, but also VB_6_ was also confirmed to be involved in methionine cycle (Franco et al., 2022). Importantly, previous study reported that VB_6_ were positively associated with hepatic steatosis, as well as correlated with triglycerides, glucose, ALT and BMI (Ferro et al., 2017). For this reason, we hypothesize that VB6 may play as the major co-factor in OCM cycle (in patients with long-term VPA treatment). Further study and clinical validation are required to explore the implications of VPA treatment on OCM-related nutrients.

Hcy, an intermediate product of the methionine cycle in OCM, is an essential amino acid derived from dietary proteins (Kaplan et al., 2020). Previous study indicated that Hcy concentration was associated with the progression of liver disease (Chien et al., 2016). In this study, ABLF patients showed a higher level of Hcy than that in NLF patients. Meanwhile, Hcy level exhibited a significant positive correlation (r = 1.334, *p* = 0.030) with VPA-associated liver dysfunction. However, the underlying mechanism for increased Hcy concentration remain elusive. In fact, clinical trials confirmed that low levels of FA and VB_12_ may lead to elevated levels of Hcy (Apeland et al., 2002; Sener et al., 2006). Moreover, serum Hcy concentration is not only mediated by nutrient levels in OCM, but also regulated by OCM-related genetic polymorphisms. Specifically, the *MTHFR* C677T variant contributed to increased serum Hcy levels in VPA-treated patients (Vurucu et al., 2008). Meanwhile, numerous studies have indicated that the *MTHFR* A1298C and *MTRR* A66G variants are related to Hcy concentrations (Zidan et al., 2013; Li et al., 2020). In this study, we found that *MTHFR* A1298C (AC, OR: 1.342, 95% CI: 1.070–1.684; CC, OR: 3.709, 95% CI: 1.088–12.637) and *MTHFR* C677T (TT, OR: 1.587, 95% CI: 0.926–2.720) variants were linked to increased Hcy levels and contributed to the development of VPA-induced liver dysfunction.

Oxidative stress is a critical pathophysiological inducer of VPA-induced liver diseases (Ma et al., 2019; Ma et al., 2020). Hcy was reported to modulate oxidative stress via regulating the levels of GSH (Perna and De Santo, 2003). In this study, we found that the VPA concentration was significantly correlated with Hcy (r = 0.4387, *p* = 0.0001) concentration. Moreover, Hcy levels exhibited a strong correlation with GSH (r = −0.6065, *P* < 0.0001) and TBARS (r = 0.6564, *P* < 0.0001) levels in patients receiving VPA-based therapy, which is consistent with previous study (Chien et al., 2016). Based on these findings, we hypothesize that 1C dysmetabolism and increased Hcy levels may be involved in the progression of VPA-induced liver dysfunction via the oxidative stress pathway.

Finally, the limitations of this study should also be discussed. First, due to the low occurrence of liver toxicity during VPA therapy (1/600-1/800 in pediatric patients, 1/20,000 in the general population) (Perucca, 2002). The sample size in this study is not adequate (45 patients in ABLF group, 112 patients in NLF group). Second, other factors (such as environmental conditions, daily living habits and diet) that may also contribute to VPA-induced liver dysfunction, which were not excluded from this cross-sectional study.

In conclusion, this study demonstrated that the *MTHFR* A1298C and *MTHFR* C677T polymorphisms as well as increased serum Hcy levels contributed to the progression of VPA-induced liver dysfunction in epileptic patients via oxidative stress pathway.

## Data Availability

The datasets presented in this study can be found in online repositories. The names of the repository/repositories and accession number(s) can be found below: China National Genebank (CNGB, https://db.cngb.org/cnsa/). The accession code is CNP0005334.
