# Supplementary material for: Associations between one-carbon metabolism and valproic acid-induced liver dysfunction in epileptic patients
Source: Front Pharmacol. 2024 Feb 23;15:1358262. doi: 10.3389/fphar.2024.1358262 (PMC10924308; doi:10.3389/fphar.2024.1358262)
Supplement: Supplementary file 1 [file Table1.DOCX]

Supplementary Table 1. List of genetic information and primer sequences for SNPs analysis.

| **Gene** | **Variant** | **Location** | **SNP ID** | **Amino acid** | **Primer sequences（5’-3’）** | **Product length** |
| --- | --- | --- | --- | --- | --- | --- |
| ***MTHFR*** | A1298C | 1p36.3 | rs 1801131 | Glu → Ala | F: CTTTGGGGAGCTGAAGGACTACTAC | 163 bp |
|  |  |  |  |  | R: CACTTTGTGACCATTCCGGTTTG |  |
| ***MTHFR*** | C677T | 1p36.3 | rs 1801133 | Ala → Val | F: TGAAGGAGAAGGTGTCTGCGGGA | 198 bp |
|  |  |  |  |  | R: AGGACGGTGCGGTGAGAGTG |  |
| ***MTR*** | A2756G | 1q43 | rs 1805087 | Asp → Gly | F: TGTTCCAGACAGTTAGATGAAAATC | 211 bp |
|  |  |  |  |  | R: GATCCAAAGCCTTTTACACTCCTC |  |
| ***MTRR*** | A66G | 5p15.31 | rs 1801394 | Ile → Met | F: GATTCAAGCCCAAGTAGT | 383 bp |
|  |  |  |  |  | R: TGCAGAAAATCCATGTAC |  |

F, forward primer; R, reverse primer.

Supplementary Table 2. Hardy-Weinberg equilibrium test and allele frequencies of four genotypes.

| **Genotypes** | **ABLF** | | | | **ABLF** | | | |
| --- | --- | --- | --- | --- | --- | --- | --- | --- |
|  | **OF** | **NF** | χ2 | ***P*** | **OF** | **NF** | χ2 | ***P*** |
| ***MTHFR* A1298C** |  |  |  |  |  |  |  |  |
| AA | 16 (35.6%) | 16.2 (36%) | 0.005 | 0.945 | 75 (67.0%) | 76.4 (68.2%) | 0.261 | 0.672 |
| AC | 22 (48.9%) | 21.6 (48%) |  |  | 35 (31.2%) | 32.2 (28.8%) |  |  |
| CC | 7 (15.5%) | 7.2 (16%) |  |  | 2 (1.8%) | 3.4 (3.0%) |  |  |
| Allele | A:54 (60.0%); C:36 (40.0%) | |  |  | A:185 (82.6%); C:39 (17.4%) | |  |  |
| ***MTHFR* C677T** |  |  |  |  |  |  |  |  |
| CC | 13 (28.9%) | 14.5 (32.2%) | 0.280 | 0.571 | 50 (44.6%) | 53.6 (47.9%) | 1.728 | 0.471 |
| CT | 25 (55.6%) | 22.1 (49.1%) |  |  | 55 (49.1%) | 47.8 (42.6%) |  |  |
| TT | 7 (15.6%) | 8.4 (18.7%) |  |  | 7 (6.3%) | 10.6 (9.5%) |  |  |
| Allele | C:51 (56.7%); T:36 (43.3%) | |  |  | C:155 (69.2%); T:36 (30.8%) | |  |  |
| ***MTR* A2756G** |  |  |  |  |  |  |  |  |
| AA | 29 (64.4%) | 28 (62.2%) | 0.133 | 0.696 | 76 (67.9%) | 73.8 (65.9%) | 0.588 | 0.568 |
| AG | 13 (28.9%) | 15 (33.4%) |  |  | 30 (26.8%) | 34.2 (30.5%) |  |  |
| GG | 3 (6.7%) | 2 (4.4%) |  |  | 6 (5.3%) | 4 (3.6%) |  |  |
| Allele | A:71 (78.9%); G:36 (21.1%) | |  |  | A:182 (81.2%); G:36 (18.8%) | |  |  |
| ***MTRR* A66G** |  |  |  |  |  |  |  |  |
| AA | 17 (37.8%) | 18.0 (40%) | 0.147 | 0.857 | 42 (37.5%) | 46.2 (41.3%) | 2.408 | 0.598 |
| AG | 23 (51.1%) | 20.9 (46.4%) |  |  | 60 (53.6%) | 51.5 (46%) |  |  |
| GG | 5 (11.1%) | 6.1 (13.6%) |  |  | 10 (8.9%) | 14.3 (12.7%) |  |  |
| Allele | A:57 (63.3%); G: 33 (36.7%) | |  |  | A: 144 (64.2%); G: 80 (35.8%) | |  |  |

OF, observed frequency; NF, expected frequency.

Supplementary Table 3. Effects of OCM-related SNPs on Hcy, Folate and Vitamin B_12_ levels in patients with epilepsy.

| **Items** | **Homocysteine** | **Folate** | **Vitamin B12** |
| --- | --- | --- | --- |
| ***MTR A2756G*** |  |  |  |
| AA (n = 105) | 7.71 ± 2.24 | 18.51 ± 3.64 | 437.56 ± 165.60 |
| AC (n = 43) | 7.79 ± 1.61 | 18.44 ± 2.88 | 408.39 ± 172.79 |
| CC (n = 9) | 7.68 ± 2.09 | 18.38 ± 2.80 | 379.06 ± 175.96 |
| *P* value | 0.976 | 0.990 | 0.436 |
| ***MTRR A66G*** |  |  |  |
| CC (n = 59) | 7.65 ± 1.84 | 18.61 ± 3.24 | 410.23 ± 158.20 |
| CT (n = 83) | 7.76 ± 2.27 | 18.41 ± 3.53 | 451.32 ± 165.57 |
| TT (n = 15) | 7.90 ± 1.85 | 18.29 ± 3.35 | 440.89 ± 201.82 |
| *P* value | 0.908 | 0.923 | 0.655 |

The bolded data indicated *P* < 0.05.

Statistical significance was determined by ANOVA.
